# Effects of the Interception of Litterfall by the Understory on Carbon Cycling in Eucalyptus Plantations of South China

**DOI:** 10.1371/journal.pone.0100464

**Published:** 2014-06-24

**Authors:** Long Yang, Jun Wang, Yuhui Huang, Dafeng Hui, Meili Wen

**Affiliations:** 1 Centre of Resource and Environment, Guangzhou Institute of Geography, Guangzhou, China; 2 Key Laboratory of Vegetation Restoration and Management of Degraded Ecosystems, South China Botanical Garden, Chinese Academy of Sciences, Guangzhou, China; 3 Guangdong Academy of Forestry, Guangzhou, China; 4 Department of Biological Sciences, Tennessee State University, Nashville, Tennessee, United States of America; DOE Pacific Northwest National Laboratory, United States of America

## Abstract

For the purposes of forest restoration, carbon (C) fixation, and economic improvement, eucalyptus (*Eucalyptus urophylla*) has been widely planted in South China. The understory of eucalyptus plantations is often occupied by a dense community of the fern *Dicranopteris dichotoma*, which intercepts tree canopy leaf litter before it reaches the ground. To understand the effects of this interception of litterfall on C cycling in eucalyptus plantations, we quantified the mass of intercepted litter and the influences of litterfall interception on litter decomposition and soil respiration. The total mass of *E. urophylla* litterfall collected on the understory was similar to that collected by the traditional litter trap method. All of the eucalyptus litterfall is intercepted by the *D. dichotoma* canopy. Of the litterfall that was intercepted by *D. dichotoma*, 20–40% and 60–80% was intercepted by the top (50–100 cm) and bottom (0–50 cm) of the understory canopy, respectively. Intercepted litterfall decomposed faster at the bottom of understory canopy (at the base of the plants) than at the top, and decomposition was slower on the soil surface in the absence of understory than on any location in the understory canopy. Soil respiration was highest when both the understory and litter were present and was lowest when both the understory and litter were absent. These results indicate that litterfall interception changed carbon flow between aboveground and belowground through litter decomposition and soil respiration, which changed carbon cycling in eucalyptus plantations. The effects of the understory on litter decomposition and soil respiration should be considered in ecosystem carbon models.

## Introduction

Forests play an important role in terrestrial carbon (C) cycling [1], [2], [3]. Because of deforestation, the forest C stock in China had decreased by 0.62 Pg by the 1970s [4]. Since then, long-term forest restoration has increased C sequestration in China [5], [6], and the establishment of plantations has increased the forest C stock by 0.45 Pg C and at a rate of 0.021 Pg C yr^−1^ [4]. China has more artificial plantations than any other country [7]. Presently, artificial plantations occupy 3379 ha and represent 32% of the forested area in China. In Guangdong Province in South China, for example, the forest cover increased from 26% in 1979 to 50% in 1998 because of artificial vegetation restoration. These plantations are considered to be important C pools that may influence climate change at the regional scale [8].

The eucalyptus *Eucalyptus urophylla*, which is native to Australia, has become a widely planted and economically important tree in South China because it grows rapidly and has other desirable characteristics. By 2010, eucalyptus plantations in South China occupied approximately 2.6 million ha [9] and were considered to be an important C sink [10]. In eucalyptus plantations, the understory vegetation is often dominated by the fern *Dicranopteris dichotoma*. Although the understory vegetation accounts for only a small portion of the total plantation biomass, it plays an important role in nutrient cycling and total production of forest ecosystems. Because of its higher nutrient content and faster biomass turnover [11], understory vegetation is considered to be a driver in forest ecosystems [12]; the understory affects many processes, such as tree seedling establishment [13], [14], litter decomposition [15], [16], and soil respiration [17],[18].

*D. dichotoma* (Gleicheniaceae) is a heliophyte fern that usually forms a dense single-species layer under the canopy of the trees in eucalyptus plantations because of its vigorous clonal growth [19] and allelopathic effects [20], [21], [22]. In eucalyptus plantations, most litter falling from the canopy is intercepted by the *D*. *dichotoma* foliage before it reaches the ground. Litter is regarded as the most important C source of forest soils. Litterfall interception may change the spatial distribution of litterfall, which would affect litter decomposition and soil respiration and thus C cycling in forest ecosystems. However, most previous studies have assumed that canopy-derived litter falls directly to the forest floor. The effect of litterfall interception by the understory C carbon cycling has not been carefully investigated.

To better understand the effects of litterfall interception by the understory vegetation, we conducted three experiments in three 6-year-old experimental eucalyptus plantations in South China. In these experiments, we determined: (1) the total mass and proportion of eucalyptus litterfall intercepted by the understory fern *D. dichotoma*; (2) the effect of litterfall interception on the rate at which the litterfall decomposes; and (3) the effect of litterfall interception on soil respiration.

## Materials and Methods

### Site description

The study was conducted at the Heshan National Field Research Station of Forest Ecosystem (112 °50'E, 22 °34'N), which is located near Heshan, Guangdong, China. All necessary permits for the field experiments were obtained from the South China Botanical Garden, Chinese Academy of Sciences. This station is one of the core stations of the Chinese Ecological Research Network (CERN) and occupies 40 ha. The location is characterized by south subtropical monsoon climate, and the soil is laterite. There is a distinctive wet season (from April to September) and dry season (from October to March). The mean annual temperature is 22.6°C, and the mean annual precipitation is 1700 mm. The annual solar radiation is 4350.5 MJ m^−2^.

As a result of long-term and severe human disturbance, the soil in the area of the station had been eroded, and the original vegetation had almost disappeared before the station was established in 1983. With the establishment of the station, the degraded land was planted with many native and exotic species as part of restoration research. The native tree species included *Castanopsis hystrix*, *Liquidambar formosana*, and *Michelia macclurei*, and the exotic tree species include *E. urophylla* and *Acacia crassicarpa*. The climax plant community in this region is subtropical monsoon evergreen broad-leaved forest and includes members of the *Lauraceae*, *Euphorbiaceae*, and *Fagaceae*.

### Mass and proportion of litterfall interception (experiment 1)

**Figure 1:**
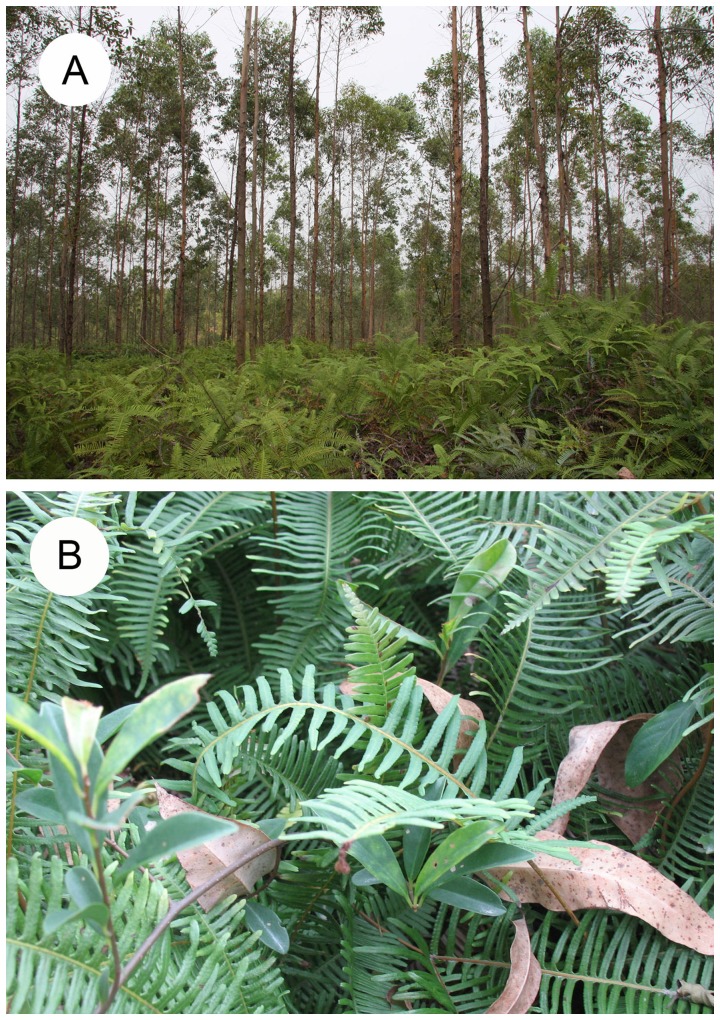
A *Eucalyptus urophylla* plantation with a dense understory of the fern *Dicranopteris dichotoma* in South China (A) and litterfall interception by *D. dichotoma* (B).

In this study, three 6-year-old forest sites were selected in which *E. urophylla* was the dominant tree and a dense community of *D. dichotoma* dominated the understory (Fig. 1A). Each site occupied ca. 1 ha, and adjacent sites were separated by ca. 100 m. The diameter at breast height of *E. urophylla* was 10.11±1.54 cm, and the height of *D. dichotoma* was 108±13 cm in 2011. The intercepted litterfall was quantified by establishing nine plots (1 m×1 m each) with three treatments and three replicates at each of the three field sites. The three treatments are referred to as litter trap (LT), open *D. dichotoma* (OD), and litter baffle (LB). The LT treatment used the traditional litter trap method. In the LT treatment, all understory *D. dichotoma* was cleared, and litter was collected using a nylon net (1 m×1 m), which was held horizontally at 100 cm above the soil surface (the average height of *D. dichotoma*) by four 1-m-high PVC tubes (4 cm diameter). The litter collected in the LT treatment can be used to estimate the total litterfall without interception by the understory. In the OD treatment, litter was collected by hand from the foliage of the *D. dichotoma* in a 1 m×1 m area. Litter in the OD treatment could move horizontally and vertically. In the LB or litter baffle treatment, litter was collected from the foliage of the *D. dichotoma* in a 1 m×1 m area as with the OD treatment, but the area was enclosed with PVC boards, which extended from the soil surface to 1 m above the soil surface. The boards or baffles prevented horizontal movement of the litter. The litter in all plots was collected every 2 months from May 2011 to May 2012. At the time of collecting, the litter in the OD and LB plots was separated into two groups according to height in the understory (0–50 cm and 50–100 cm, as measured from the ground). All litter was weighed after it had been oven-dried at 65°C for 72 h.

### Litter decomposition (experiment 2)

Litter decomposition was quantified in three plots (1 m×1 m each) at each field site. Fresh litter of *E*. *urophylla* was collected with litter traps, air-dried for 15 days, and then added to 10 cm×10 cm litter bags (10 g of air-dried litter per bag). The mesh size on the upper side of the nylon litter bags was 0.8 mm to access the soil organisms and the mesh size on the bottom side was 0.25 mm to prevent litter loss. Thirty litter bags were placed in each plot at different heights: 10 were placed on the top of the *D. dichotoma* canopy (ca. 100 cm above the forest floor); 10 were placed at mid-height of the *D. dichotoma* canopy (ca. 50 cm above the forest floor); and 10 were placed on the *D. dichotoma* roots that grew on the surface of the forest floor. Another 10 litterbags were placed directly on the ground in an area where all of the *D. dichotoma* had been removed. One litterbag per treatment per plot was retrieved every 2 months from April 2011 to June 2012. After recovery, the litterbags were opened, and the decomposing litter was carefully removed and cleaned of adhering soil particles. The litter was then oven-dried at 65°C to constant mass and weighed. The *E*. *urophylla* litter decomposition rate (decay constant, *k*) was determined with a negative exponential decay model [23], [24]: *y* = *e* ^–*kt*^, where *y* is the proportion of the initial biomass remaining at time *t* (yr^−1^), and *k* is the litter decay constant. The model was fitted by nonlinear regression in Sigmaplot 12.0 (Systat Software, San Jose, CA).

### Soil respiration (experiment 3)

This experiment tested the effects of litterfall and the understory fern on soil respiration. In each of the three field sites, three blocks were established. Each block (2 m×2 m) included four 1 m×1 m plots to accommodate four treatments (two levels of each of two factors, i.e., + understory fern and + litter). The spatial separation of blocks and plots were about 10 m and 20 cm, respectively. The four treatments were: 1) minus understory fern and minus litter (-D-E); 2) minus understory fern and plus litter (-D+E); 3) plus understory fern and minus litter (+D-E); and 4) plus understory fern and plus litter (+D+E). The plus treatments involved complete retention of the fern or litter, and the minus treatments involved complete removal of the fern or litter (removal was done each week to maintain the treatments). The ferns were uprooted by hand, and the disturbance from this treatment was greatly reduced, for the root of the fern was shallow and the experiment started after 2 months of treatment. In the +E treatments, the litter was placed on the surface roots at the base of the fern. In each plot, one soil collar (PVC pipe, 10 cm in diameter and 5 cm in height) was set up in the soil (2 cm deep) for soil respiration measurement. In total, there were 12 collars (3 plots×4 treatments) at each site. Soil respiration rate was measured twice each month from April 2011 to March 2012 with a soil chamber (Li-6400-09) connected to a Li-6400 portable gas exchange analyzer (Li-Cor, Biosciences, Lincoln, Nebraska, USA). All measurements of soil respiration were conducted between 09:00 and 14:00 each time with no precipitation, and three measurements were taken at each sampling point. Soil temperature was recorded simultaneity by soil temperature probe that connected to the Li-6400. Soil humidity was measured by a time domain reflectometry (TDR, TRIME-FM, IMKO, Ettlingen, Germany). The regression relationship between soil respiration and soil temperature were fitted by exponential growth model: 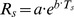, where *R_s_* is the soil respiration rate; *T_s_* is soil temperature at 5°C depth; *a* and *b* are fitted constants. *Q_10_* was calculated as 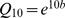. The regression relationship between soil respiration and soil humidity were fitted by linear model: 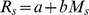, where *M_s_* is soil humidity at 5°C depth; *a* and *b* are fitted constants.

### Statistical analysis

For experiment 1, a three-way analysis of variance (ANOVA) was used to determine the effects of time, site, treatment, and their interactions on litterfall interception by the understory fern *D. dichotoma*; in addition, a three-way ANOVA was used to determine the effects of time, site, height in the *D. dichotoma* understory, and their interactions on litterfall interception in the OD and LB treatments. For experiment 2, one-way ANOVAs were used to determine the effect of height in the understory and absence of understory on mass remaining in litter bags at the end of the experiment and on the decomposition decay constant. For experiment 3, four-way ANOVAs were used to test the effects of season (wet or dry), site, litter (+), understory (+), and their interactions on soil respiration. When an effect was significant at α = 0.05, means were compared by least significant difference (LSD) tests with SYSTAT 11.0 (Systat Software Inc., USA). Graphs were made with Sigmaplot 9.0 (Systat Software, Inc., Point Richmond, CA, USA).

## Results

### Mass and proportion of litterfall intercepted by the understory (experiment 1)

**Figure 2:**
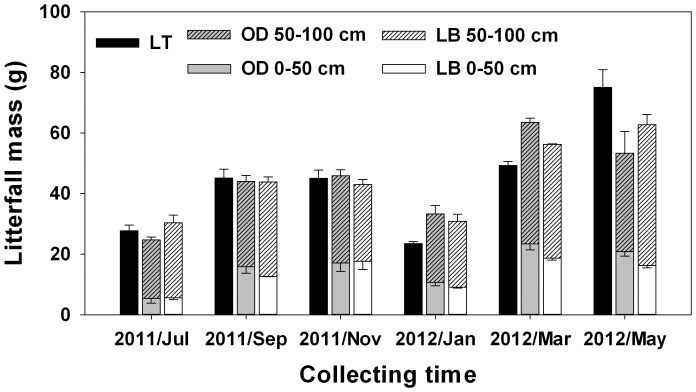
Mass of litterfall intercepted (means ± SE) by traditional litter traps (LT), the natural *Dicranopteris dichotoma* understory (OD), and the *D. dichotoma* understory with bordering baffles to prevent horizontal movement of litter (LB) (experiment 1). The mass of litter captured in LT can be considered equivalent to the total mass of tree litter that can potentially fall to the forest floor.

The mass of litterfall was unaffected by treatment, site, or the interactions but was affected by time (Table 1). The largest amount of leaf litter was collected in May 2012, and the smallest amount was collected in January 2012 (Fig. 2). For OD and LB treatments, litter interception was significantly affected by height in the understory (0–50 cm or bottom vs. 50–100 cm or top) and time (Table 2). For the OD treatment, more leaf litter was collected from the bottom (61–80% of the total) than from the top (22–39%) of the *D. dichotoma* patch (Fig. 2). For the LB treatment, more leaf litter was collected from the bottom (59–81%) than from the top (19–41%) of the *D. dichotoma* patch.

**Table 1:** Results of ANOVA for the effects of time (date of litter collection), site, and treatment (LT, OD, or LB) on the total mass of intercepted litterfall (experiment 1).

Source	*df*	F	*P*
**Time**	5	35.488	**<0.001**
**Site**	2	1.819	0.174
**Treatment**	2	0.013	0.987
**Time×Site**	10	0.133	0.999
**Time×Treatment**	10	0.502	0.879
**Site×Treatment**	4	0.298	0.878
**Time×Site×Treatment**	20	0.064	1.000

Note: LT refers to litter collected on nylon sheets (litter traps); OD refers to litter collected on natural *D. dichotoma* foliage; LB refers to litter collected on *D. dichotoma* foliage with baffles on the plot borders that prevented the horizontal movement of the litter.

**Table 2:** Results of ANOVAs for the effects of time (date of litter collection), site, and height (position in the understory canopy: from 0–50 vs. 50–100 cm from the ground) on the total mass of litter intercepted by the canopy in the OD treatment (litter collected on natural *D. dichotoma* foliage) and the LB treatment (litter collected on *D. dichotoma* foliage with baffles on the plot borders that prevented the horizontal movement of litter) (experiment 1).

Source	*df*	OD		LB	
		F	*p*	F	*p*
**Time**	5	18.999	**<0.001**	11.030	**<0.001**
**Site**	2	6.185	**0.006**	0.483	0.622
**Height**	1	104.106	**<0.001**	122.837	**<0.001**
**Time×Site**	10	0.111	1.000	0.053	1.000
**Time×Height**	5	0.054	0.998	0.588	0.709
**Site×Height**	2	0.130	0.878	0.054	0.947
**Time×Site×Height**	10	0.143	0.999	0.045	1.000

### Litter decomposition (experiment 2)

**Figure 3:**
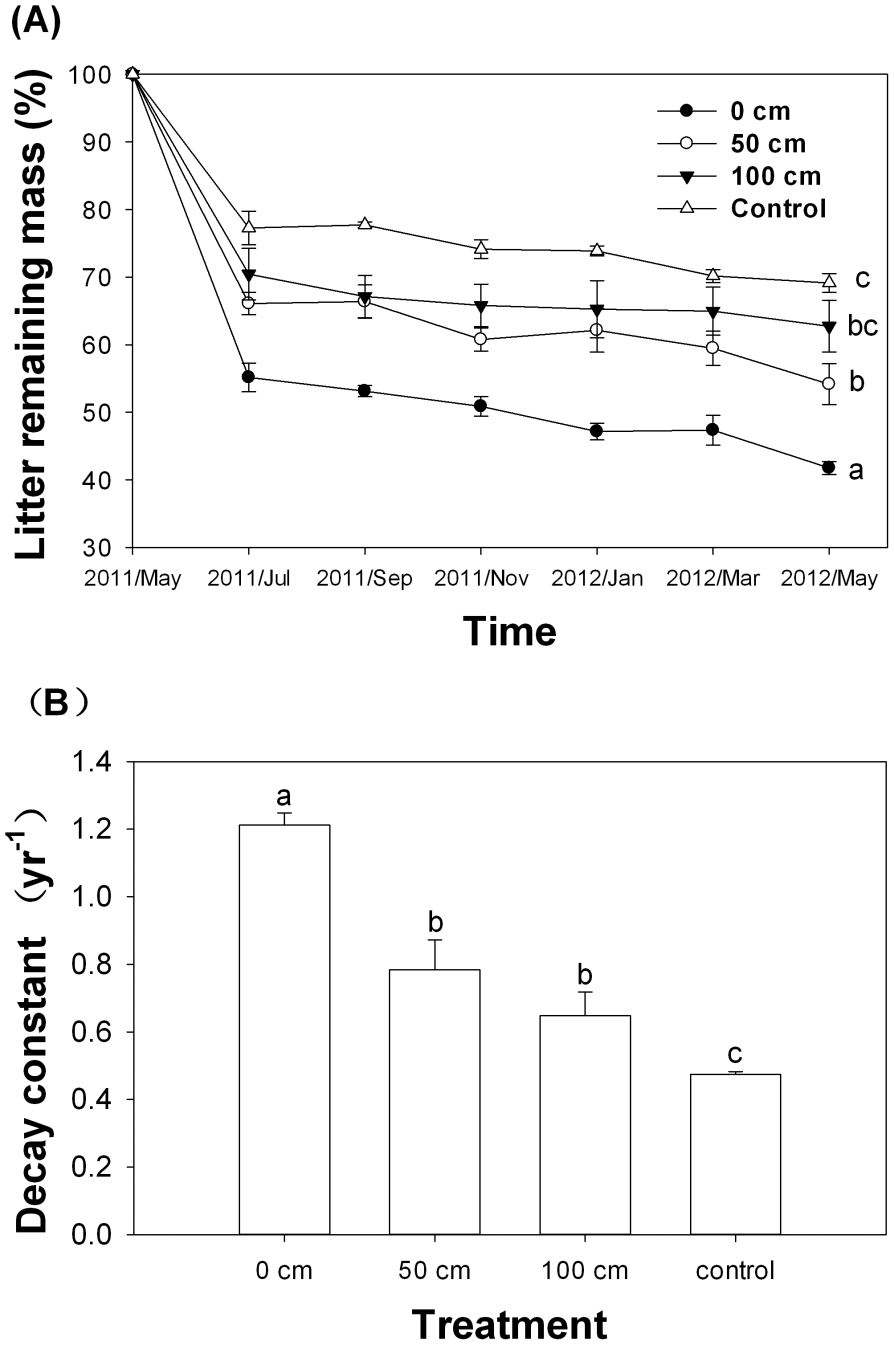
Litter mass remaining (A) and litter decomposition rate (*k*) (B) at different heights (0, 50, and 100 cm) in the *Dicranopteris dichotoma* understory or on the bare ground without understory (control) (experiment 2). Error bars represent standard errors, and different letters indicate significant differences at *α* = 0.05.

The litter mass remaining in all treatments followed a typical litter decomposition pattern and was always greater for litter bags kept in the *D. dichotoma* canopy (at 0, 50, or 100 cm) than on the surface of the soil in an area without understory (control) (Fig. 3A). Litter decomposition was the highest during the first 2 months of the experiment. At the end of the experiment, the litter mass remaining differed among the treatments (F = 13.513, *p* = 0.006). At the end of the experiment, litter mass remaining was 41.8, 54.2, and 62.7% at 0 cm, 50 cm, 100 cm height, respectively, in the *D. dichotoma* canopy and was 69.1% on the bare ground without *D. dichotoma*. The litter decomposition rate (decay constant, *k*) declined with distance from the ground to canopy (*p* = 0.003, F = 17.803, *df* = 2) and was lowest on the bare ground without *D. dichotoma* (Fig. 3B).

### Soil respiration (experiment 3)

**Figure 4:**
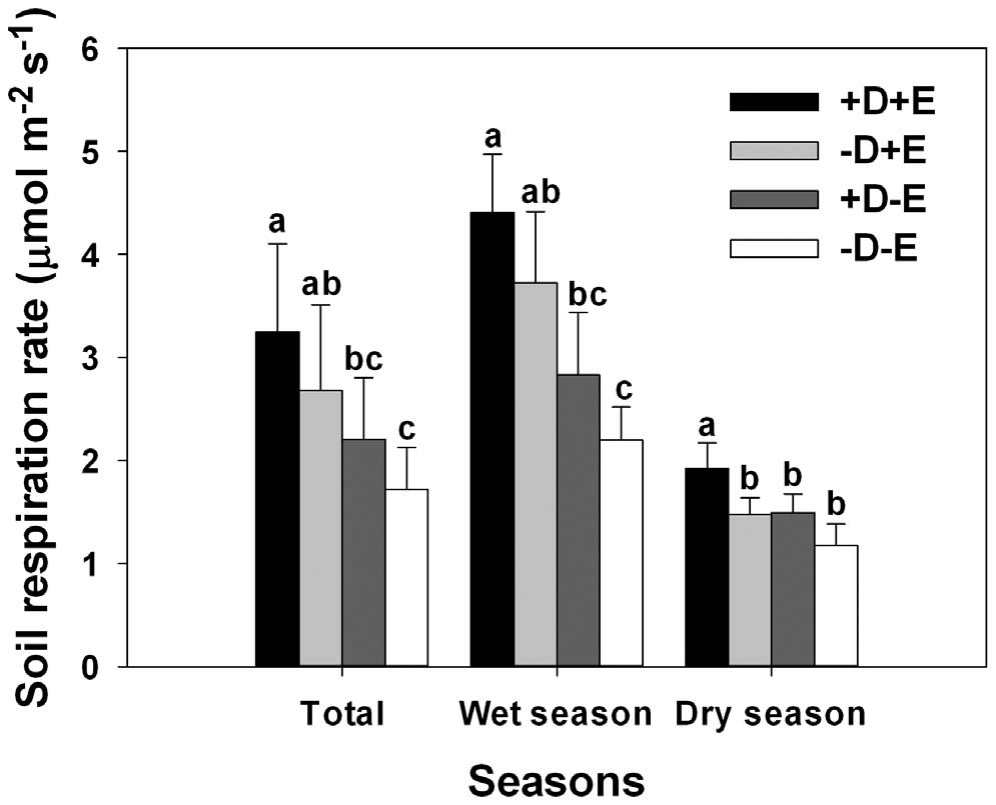
Soil respiration rate as affected by treatment (-D-E, -D+E, +D-E, and +D+E) and season (wet and dry season) in *Eucalyptus urophylla* plantations (experiment 3). D refers to the *Dicranopteris dichotoma* understory, which was present or absent (+). E refers to tree litter, which was present (at the base of *D. dichotoma* or on bare ground) or absent (+). Different letters above bars indicate significant differences (at *α* = 0.05) among the treatments in the same season. Error bars represent SEs.

**Figure 5:**
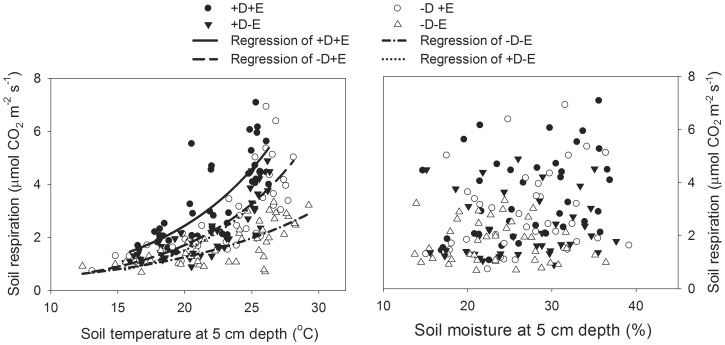
Relationship between soil respiration rate and soil temperature (A) or soil humidity (B) under all treatments: -D-E, -D+E, +D-E, and +D+E.

Season (wet vs. dry), litter (+), understory (+), and the interaction between season and litter significantly affected soil respiration (Table 3). Regardless of litter or understory treatment, the soil respiration rate was much higher in the wet season (from April to September) than in the dry season (from October to March) (Fig. 4). In the dry season, soil respiration was higher in the +D+E treatment than in the other three treatments (Fig. 4). In the wet season and over the entire experiment, the treatments were in the following order with respect to soil respiration rates: +D+E>-D+E>+D-E>-D-E (Fig. 4). The relationship between soil respiration rate and soil temperature was well fit by an exponential growth regression model (*p*<0.0001). Soil temperature explained 40%, 48%, 38% and 27% of the variations in +D+E, +D-E, -D+E and–D-E treatments, respectively (Fig. 5, Table 4). *Q_10_* was ranged from 2.45 to 4.38, and the highest value appeared in +D-E treatment. Soil humidity have no linear relationship with soil respiration (*p*>0.05 in all treatments).

**Table 3:** Results of ANOVA for the effects of season (wet vs. dry), site, litter (+), understory (+), and their interactions on the soil respiration rate.

Source	*df*	F	*p*
**Season**	1	126.216	**0.000**
**Site**	2	0.810	0.447
**Litter**	1	40.766	**0.000**
**Understory**	1	11.838	**0.001**
**Season×Site**	2	1.055	0.351
**Season×Litter**	1	12.002	**0.001**
**Site×Litter**	2	0.294	0.746
**Season×Site×Litter**	2	0.064	0.938
**Season×Understory**	1	0.565	0.453
**Site×Understory**	2	0.235	0.791
**Season×Site×Understory**	2	0.003	0.997
**Litter×Understory**	1	0.159	0.691
**Season×Litter×Understory**	1	0.029	0.866
**Site×Litter×Understory**	2	0.840	0.434
**Season×Site×Litter×Understory**	2	0.390	0.678

**Table 4:** Effects of soil temperature (*T_s_*) and soil moisture (*M_s_*) on the variation of soil respiration rate (*R_s_*) under all treatments (-D-E, -D+E, +D-E, and +D+E).

Regression	Treatment	*p*	*R^2^*	*a*	*b*	*Q_10_*
***R_s_*** ** & ** ***T_s_***	-D-E	<0.0001	0.2661	0.2091	0.0898	2.45
	-D+E	<0.0001	0.3777	0.1295	0.1292	3.64
	+D-E	<0.0001	0.4787	0.0791	0.1479	4.38
	+D+E	<0.0001	0.3988	0.1995	0.1254	3.50
						
***R_s_*** ** & ** ***M_s_***	-D-E	0.474	0.0002	−0.0166	2.1563	-
	-D+E	0.107	0.0047	0.0676	1.0099	-
	+D-E	0.779	0.0000	0.0088	2.0629	-
	+D+E	0.053	0.0095	0.0801	1.3011	-

The regression relationship between *R_s_* and *T_s_* (*R_s_* & *T_s_*) was fitted by exponential growth model 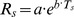, 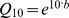; The regression relationship between *R_s_* and *M_s_* (*R_s_* & *M_s_*) was fitted by linear model 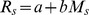.

## Discussion

### Mass of litterfall

Litterfall interception is greatly influenced by plant architecture (arrangement of branches and leaves) and morphology (stalks, spines, and leaf size) [25]. *D. dichotoma* propagates with spores or clones and always forms a dense understory [20]. The aboveground biomass of *D. dichotoma* ranges from 1360 to 3411 kg ha^−1^, and the understory canopy cover can be as high as 100% [26]. *D. dichotoma* intercepts litterfall from tree species in its interlaced fronds and stalks. Because *E*. *urophylla* leaves are relatively large [27] and light [28], they are readily intercepted and supported by the *D. dichotoma* canopy (Fig. 1B). The results of our first experiment indicated that virtually all of the eucalyptus litterfall is intercepted by the *D. dichotoma* canopy because the quantity of litterfall collected from the understory canopy was similar to that collected by traditional traps, which presumably capture nearly 100% of the litterfall. The quantity of intercepted litterfall was also reported to be substantial in other ecosystems [25], [31], and we quantified the mass of litterfall interception by the fern and compared litterfall collected using different methods. Our results indicated that the OD and LB methods provided reliable estimation of litterfall. In the current study, the annual litterfall in the eucalyptus forests was about 2.657 Mg ha^−1^. This value was comparable to those in previous studies in eucalyptus forests [29], [30].

Litterfall interception in our study also varied in time and space. With regard to time, litterfall interception (and total litterfall) was highest from March to May (i.e., between the dry and wet season) and was lowest in the wet season (from May to July). With regard to space, more litterfall was intercepted at the bottom (0–50 cm height) than at the top of the understory (50–100 cm height).

### Effects of litterfall interception on litter decomposition

Litter decomposition is influenced by the physicochemical environment, litter quality, and the composition of the decomposer community [31]. Litter quality was excluded as a variable in our decomposition experiment current study because the litter that was placed in the litter bags was identical. Decomposition, however, can be influenced by the understory vegetation because the understory can affect the location of litter [25] and therefore affect the decomposition microenvironment. Litter decay was reduced when litterfall was intercepted by the *D. dichotoma* canopy, probably because of spatial heterogeneity of physicochemical environment and the composition of the decomposer community in the understory fern. Humidity may be a more important factor which can significantly affects microbial composition and activity and litter decay rate [32], [33]. For example, in *Cinnamomum camphora* plantation forest in South China, litterfall interception of understory vegetation delayed the litter decomposition, for water content on the crown may inhibited microbial activities [34]. Although light could also change microbial community characteristics and affect litter decomposition, it may not be an important factor in this study, as litter decomposition controlled by photodegradation happened in arid or semi-arid ecosystem [35], not in subtropical ecosystem. The composition of the decomposer community could change the litter decay rate, but we did not determine the composition of the decomposer community.

We also found that the litter decayed more quickly in the *D. dichotoma* canopy than on bare soil without canopy. This result was consistent with Dearden and Wardle [25], who reported that litter of canopy species in the crowns of the understory ferns decayed faster than that on the ground. In another study, O'Connell [16] found that the understory legume *Acacia pulchella* could increase the decomposition rate of *E. marginata* canopy litter. The results of this study also indicate that the overall litter decomposition rate of tree leaf litter in forests with understory may be underestimated when litter bags are places on bare ground in the absence of understory.

### Effects of litterfall interception on soil respiration

Soil respiration includes autotrophic respiration from root and heterotrophic respiration from microbe and soil fauna. Soil respiration showed obvious seasonal variation, higher in wetter season and lower in drier season, which affected by south subtropical monsoon climate. Warmer temperature and abundant water content may increase the activity of microbe and fauna. In our study, both key factors (understory vegetation and litter) may influence soil respiration. Our results concerning the effects of the understory on soil respiration are consistent with several previous studies. In the same eucalyptus forests of South China, for example, removal of understory vegetation reduced annual soil respiration by 6% [35]. In a deciduous broad-leaved forest, removal of understory dwarf bamboo (*Sasa senanensis*) also reduced soil respiration [36]. In the current study of eucalyptus forests, removal of litter reduced soil respiration by 36%. The reported contributions of litter to soil respiration in artificial and natural forests in China and other countries have ranged from 12 to 41% [17], [35], [37], [38], [39], [40], [41], [42]. Soil respiration rate was higher in the +D-E treatment than in the -D-E treatment, indicating that the understory substantially affected soil respiration even in the absence of litter. This can be attributed to respiration by *D. dichotoma* roots, which are shallow and abundant; root biomass is usually higher than aboveground biomass for *D. dichotoma* [26].

Soil temperature and soil humidity are the most important environmental factors, which influence the soil respiration. However, the relationship varied along with spatio-temportal difference. Some study found soil respiration was just related with soil temperature or soil humidity [43], or soil respiration was related with soil temperature and soil humidity [44], [45]. Our results, that soil respiration was just related with soil temperature and had no relationship with soil humidity, were different from previous studies in South China [35]. Lower soil porosity may impede CO_2_ release when soil humidity increase. In climax vegetation of South China, subtropical monsoon evergreen broad-leaved forest, both of soil temperature and soil humidity affected soil respiration, and soil humidity had greater influence [46]. While in artificial plantations of monocultures, lower canopy cover, higher sun radiation of understory layer and weaker buffer of air temperature led to spatial heterogeneity of soil temperature and microbial activity, which may be the reason why soil respiration was sensitive to soil temperature. Under global warming condition, the temperature sensitivity of soil respiration has become the focus topic and *Q_10_* was widely used [47], [48]. Most studies found that *Q_10_* was ranged from 1.3 to 3.3, and decreased with latitude reducing [49]. Higher *Q_10_* in Eucalyptus plantations (>2.4), means that more CO_2_ will be released from soil respiration in the future, especially when litterfall were intercepted by understory fern (+D-E treatment, *Q_10_* = 4.38).

### Effects of litterfall interception on carbon cycling

Litterfall interception by the understory is a ubiquitous phenomenon in subtropical and tropical forests because the understory in such forests is always dense. In this study, which was conducted in eucalyptus plantations in South China, we quantified the vertical distribution of litterfall in the understory, which was dense and dominated by the fern *D. dichotoma*, and tested the effects of litterfall interception on litter decomposition and soil respiration. Although heterogeneous soil structure appeared, the results from smaller plots measured in our study for even plantation landscape indicate that litter interception by the understory fern *D. dichotoma* changes two important processes of carbon flow between aboveground and belowground, litter decomposition and soil respiration, which changes carbon cycling.

As part of vegetation restoration efforts, plantations now occupy large areas worldwide. Such plantations are considered to be important carbon pools that might mitigate climate change at a regional scale. Although understory vegetation acts as a transitional layer that links aboveground and belowground processes, the ecosystem functions of understory vegetation in plantations have generally been ignored because the understory biomass is much less than the tree biomass. - However, understory vegetation is an important driving force of nutrient cycling and productivity in forest ecosystems [12]. In addition to accumulating biomass, understory plants reduce litter decomposition rates and soil respiration rates and therefore enhance carbon sequestration as a consequence of litterfall interception. Because litterfall interception will affect carbon budgets and cycling, the effects of litterfall interception by understory vegetation should be considered in biogeochemical models of forest ecosystems.
